# Effectiveness of different databases in identifying studies for systematic reviews: experience from the WHO systematic review of maternal morbidity and mortality

**DOI:** 10.1186/1471-2288-5-6

**Published:** 2005-01-28

**Authors:** Ana P Betrán, Lale Say, A Metin Gülmezoglu, Tomas Allen, Lynn Hampson

**Affiliations:** 1 UNDP/UNFPA/WHO/World Bank Special Programme of Research, Development and Research Training in Human Reproduction, Department of Reproductive Health and Research, World Health Organization, Geneva, (1211) Switzerland; 2 Library & Information Networks for Knowledge, Department of Knowledge Management and Sharing, World Health Organization, Geneva, (1211) Switzerland; 3 Cochrane Pregnancy and Childbirth Group, Liverpool Women's Hospital NHS Trust, Liverpool L8 7SS, UK

## Abstract

**Background:**

Failure to be comprehensive can distort the results of a systematic review. Conversely, extensive searches may yield unmanageable number of citations of which only few may be relevant. Knowledge of usefulness of each source of information may help to tailor search strategies in systematic reviews.

**Methods:**

We conducted a systematic review of prevalence/incidence of maternal mortality and morbidities from 1997 to 2002. The search strategy included electronic databases, hand searching, screening of reference lists, congress abstract books, contacting experts active in the field and web sites from less developed countries. We evaluated the effectiveness of each source of data and discuss limitations and implications for future research on this topic.

**Results:**

Electronic databases identified 64098 different citations of which 2093 were included. Additionally 487 citations were included from other sources. MEDLINE had the highest yield identifying about 62% of the included citations. BIOSIS was the most precise with 13.2% of screened citations included. Considering electronic citations alone (2093), almost 20% were identified uniquely by MEDLINE (400), 7.4% uniquely by EMBASE (154), and 5.6% uniquely by LILACS (117). About 60% of the electronic citations included were identified by two or more databases.

**Conclusions:**

This analysis confirms the need for extending the search to other sources beyond well-known electronic databases in systematic reviews of maternal mortality and morbidity prevalence/incidence. These include regional databases such as LILACS and other topic specific sources such as hand searching of relevant journals not indexed in electronic databases. Guidelines for search strategies for prevalence/incidence studies need to be developed.

## Background

The importance of comprehensive search strategies to identify 'all relevant articles' when conducting systematic reviews has been long documented [1]. Comprehensive strategies include systematic searching of multiple databases as well as hand searching and contacting relevant experts. These strategies, however, may yield thousands of citations from which only a small number is eventually included in the review. Knowing which sources yield a reasonable number of relevant studies on a health topic may help those planning and conducting systematic reviews in that particular area.

At the World Health Organization (WHO), we have conducted a systematic review of prevalence/incidence of maternal mortality and morbidities from 1997 to 2002. The primary objective of this review is to assist in mapping the burden of reproductive ill-health by providing a comprehensive, standardized and reliable tabulation of data on the prevalence/incidence of maternal morbidity and mortality [2]. This article evaluates the usefulness of different sources in identifying data for the systematic review. We also discuss limitations of the databases and implications for future systematic reviews of observational studies.

## Methods

The methodology of the systematic review and the search strategy have been described elsewhere [3]. In brief, we searched for reports of maternal mortality and morbidity across various study designs (cross-sectional, cohort, census, RCTs, etc.). The search included multiple electronic databases: MEDLINE, Popline, EMBASE, CINAHL, CAB Abstracts, Econlit, Sociofile, LILACS, BIOSIS and PAIS International. Specialist librarians together with the reviewers developed database-specific search strategies according to the particular subject headings and searching structure of the databases (See Additional file 1). Given the importance of retrieving data from developing countries, we also identified and searched databases in developing countries such as Index Medicus of the Eastern Mediterranean Region (IMEMR) [4]; African Index Medicus (AIM) [5]; IndMED [6], a bibliographic database of Indian biomedical journals; and HELLIS.ORG [7], a network of health science libraries across Asia. However, the search for some of these databases did not yield complete results due to limited functionality of these systems (e.g. lack of essential information from the citation, inconsistencies) and their results, except for IMEMR, are not presented independently but under 'other' in Table 1. The search also included hand searching of journals not indexed in major databases and available at WHO's library, government reports, screening of reference lists of retrieved articles, congress abstract books, and contacting experts active in the field for unpublished datasets.

**Table 1:** Results of the various types of searches

Source	Number identified	Number included	Number unique included*	Sensitivity (%)	Precision (%)
MEDLINE	38986	1590	400	61.6	4.1
Popline	3255	297	91	11.5	9.1
EMBASE	23133	1135	154	43.9	4.9
CINAHL	9090	263	9	10.2	2.9
CAB Abstracts	4317	190	19	7.4	4.4
Econlit	188	4	1	0.2	2.1
SocioFile	1618	32	8	1.2	2.0
LILACS	1456	137	117	5.3	9.4
BIOSIS	3313	436	21	16.9	13.2
PAIS International	705	21	3	0.8	3.0
IMEMR	109	11	5	0.4	10.1
All Electronic databases**	**64098**	**2093**	**NA**	**81.1**	**3.3**
					
**Other^§^**	**487**	**487**	**487**	**18.9**	**NA**
***TOTAL*^±^**	***64585***	***2580***	***2580***	***NA***	***4.0***

* Refers to the number of citations that were exclusively identified by each database.

** Refers to all citations identified through the electronic databases above after duplicate entries are removed.

^§ ^Includes hand searching, contacting experts, conference proceedings, reference lists, library collections of journals, and databases in developing countries.

^± ^Refers to all citations identified through all methods after duplicate entries are removed. NA: Non-applicable

We downloaded all citations identified by electronic databases into Reference Manager^® ^software. Selection of eligible studies involved two stages; the first stage consisted of screening of title and/or abstracts from the citations downloaded. Citations were excluded if any of the following applied: (i) data collection dates were not reported, (ii) data were collected only before 1990, (iii) part of the data were collected before 1980 and disaggregation by year was not possible (in order to exclude data before 1990), (iv) number of study participants was less than 200, (v) the study design was case-control and incidence/prevalence estimates from the defined population cannot be calculated, (vi) the methodology was not described. The second stage consisted of full-text evaluation of those citations that were not excluded in the first step applying the same criteria. For practical reasons, studies identified by other sources were only entered in Reference Manager^® ^if they were eventually included in the review.

We recorded the source or sources of each citation. For each source, we calculated number of citations identified, included, and number included that were unique to the source. The sensitivity of each source was defined as the number of included citations identified by the source over the total number included. The precision was defined as the number of included citations identified by a source over the number of both included and excluded citations identified by that source. This review had no language restrictions and we recorded the language in which the report was written. Detailed results regarding languages will be reported separately.

Two reviewers performed the screening for all citations. In order to estimate the level of disagreement between the two reviewers within 2.5% of the true value, they independently screened title/abstracts for a sample of citations (560). This sample size assumes a 95% confidence interval and that the level of disagreement between the two reviewers will not exceed 10% [8]. The percentage of agreement was 88.9% (95% CI 86.0% to 91.4%). The inter-observer agreement beyond chance was calculated using the Kappa statistics and found to be 0.60 (95% CI 0.52 to 0.69). This value corresponds to moderate to substantial agreement between the reviewers.

## Results

For the time period from 1997 to 2002, 64098 different citations from electronic databases were identified of which 2093 were included. Additionally, 487 citations were included from other sources. There were 92 citations for which we could not obtain full text and, therefore, they could not be assessed (2% of those that required full-text evaluation).

Table 1 shows a breakdown of the results by source including, for each source of data, number of citations identified, number included, number included unique to each source, sensitivity and precision. Overall, electronic databases identified four fifths (81.1%) of the included citations. MEDLINE and EMBASE which identified about 62% and 44% of the included citations respectively were most sensitive. Considering electronic citations alone (2093), almost 20% were identified uniquely by MEDLINE (400), 7.4% uniquely by EMBASE (154), and 5.6% uniquely by LILACS (117). About 60% of the electronic citations included were identified by 2 or more databases.

Overall, in terms of precision, we included 1 in 25 screened citations (4%). The most precise database was BIOSIS where 13.2% of the screened citations were included; IMEMR, LILACS and Popline followed this with 10.1%, 9.4% and 9.1%, respectively (see Table 1). MEDLINE and EMBASE were similarly precise, 4.1% and 4.9%, respectively.

Preliminary analysis regarding languages revealed that about 20% of the included studies were published in other languages than English. Spanish and French were, after English, the most used languages for reporting.

## Discussion

Failure to identify relevant information in systematic reviews can result in bias [9]. The importance of including other sources of data in addition to electronic databases in general and MEDLINE in particular has been documented, especially for clinical or randomised controlled trials [1,9-11]. On the other hand, search strategies for systematic reviews of observational data on morbidities are less precise, more difficult to narrow the focus and have been studied to a lesser extent [12]. This led us to perform for our systematic review an extensive search strategy that is highly sensitive but barely precise. From 64098 citations identified only 2580 were included which represents 4% of the scrutinized articles.

Although MEDLINE identified about 62% of all citations and 76% of electronic citations relevant for this review, sources of data other than the major electronic databases are confirmed to be crucial. Some 487 (one fifth) citations were identified by reference lists of articles, expert contacts, congress proceedings, abstract books, hand searching of journals available in libraries that are not indexed in electronic databases, and other emerging databases in developing countries. As expected, there has been a large overlap between databases: 60% were identified by two or more databases and about 44% were identified by MEDLINE and EMBASE together. These two databases also provided the largest number of unique citations and both are considered necessary. PAIS International and Econlit only identified 3 and 1 citations, respectively, that were not identified by any other database and they could probably be disregarded in future reviews.

The nature of this systematic review with its focus on settings where burden of disease is highest necessitates extensive searching of developing country sources. However, literature from developing countries is difficult to access and it is not well represented in MEDLINE or other well-known electronic databases [13,14]. An editorial by Zielinksi in 1995 stated that only 2% of the journals indexed in MEDLINE or the Science Citation Index were from developing countries [15]. In 2004, the situation was similar. We calculated the number of journals published in developing countries and also indexed in MEDLINE to be about 6%. In 1996, the whole Latin American continent accounted for 0.39% of the total number of articles included in MEDLINE, down from a high of 2.03% in 1966 [16]. One of the reasons for this is the indexing of journals on a priority system where the impact factor of a journal influences its chances of being indexed. This results in country bias since western journals have in general higher impact factors, and they are therefore more likely to be indexed than those from developing countries.

The value of LILACS database to improve the quality of systematic reviews has been previously reported [17]. Our analysis confirms LILACS as a unique source of information for the Latin America and the Caribbean region that is not covered in other databases (117 unique citations included). Unfortunately, specific databases for other less developed regions like Asia and Africa, are just emerging or their access and functioning limited (e.g. AIM, IMEMR, IndMED, HELLIS.ORG). Although these regional databases are included in the review, the results are not presented individually but under 'other' in Table 1. With these databases, we experienced language barriers, difficulty in obtaining abstracts and full-text reports, inconsistencies, lack of essential information from the citation (e.g. year or title missing) and other technical problems. We believe that the low number of citations identified by IMEMR (see Table 1) is due more to the limitations mentioned above than to lack of data. These regional databases provide unique relevant citations and incomplete access limits their usefulness. Strengthening the functionality and improving the search facility of these databases could provide substantial relevant information.

A limiting factor for identifying citations is related to late indexing of journals in electronic databases. Search strategies for this review were conducted in early 2003 to identify articles published in 2002 or earlier. While only few articles published in 2002 could be expected not to be in the databases by 2003, some articles published in 1997 were only appearing in the databases as late as 2003. Traditionally, EMBASE has been found to index faster than MEDLINE, thus supporting the argument to search multiple databases [18]. Furthermore, each database producer has a particular schedule that the searcher needs to be aware of. For example, MEDLINE available through OVID, due to the updating of the MeSH terms by the National Library of Medicine, will cease entry of new citations in November and only update the database in January of the following year. These factors need to be considered in assessing the yield from different databases. It is necessary to determine how to capture these 'late indexed' citations whether by delaying the running of the search or building into follow-up studies the need to capture these citations. The electronic search for this review would have probably captured more citations had it been run in 2004.

This systematic review involved significant financial and human resources over a 3-year period [3]. Screening of a large number of citations and retrieving the full text of about 5000 articles have resource implications that need to be balanced with the benefits of the results. For this type of reviews, decisions on the extent of the comprehensiveness of the search strategy should take the resource implications into account. A careful selection of databases to be used and tailored search strategies for each database would help to maximise the benefits compared to costs.

## Conclusions

1. In contrast with RCTs, guidelines for search strategy of systematic reviews of observational studies in general, and incidence/prevalence studies in particular, need to be developed.

2. Searching beyond the major electronic databases such as MEDLINE or EMBASE is necessary to identify studies in journals from less developed countries.

3. Regional databases indexing citations from local journals not reaching MEDLINE are especially relevant for this type of systematic review. They need to be fully functioning and made worldwide accessible. A network for accessing the full text of these articles would be helpful.

## Competing interests

The author(s) declare that they have no competing interests.

## Authors' contributions

APB, LS, AMG, TA and LH developed the search strategy for the systematic review. APB and LS analysed the data and wrote the manuscript. AMG, TA and LH participated in the interpretation of results and provided key comments on the manuscript. All authors read and approved the final manuscript.

## Pre-publication history

The pre-publication history for this paper can be accessed here:



## Supplementary Material

Additional file 1 Detailed search strategy for electronic databases used for the systematic review. Click here for file
