# Supplementary material for: Effectiveness of different databases in identifying studies for systematic reviews: experience from the WHO systematic review of maternal morbidity and mortality
Source: BMC Med Res Methodol. 2005 Jan 28;5:6. doi: 10.1186/1471-2288-5-6 (PMC548692; doi:10.1186/1471-2288-5-6)
Supplement: Additional file 1 — Detailed search strategy for electronic databases used for the systematic review. [file 1471-2288-5-6-S1.doc]

**Additional file 1: Search strategy for electronic databases**

**Medline (Ovid):**

1. exp epidemiologic methods/ or comparative studies/
2. exp "OUTCOME AND PROCESS ASSESSMENT (HEALTH CARE)"/
3. sn.xs.
4. (evaluation studies or meta analysis or multicenter study).pt.
5. or/1-4
6. (maternal adj4 mortal$).af.
7. (maternal adj4 death$).af.
8. (pregnan$ adj4 death$).af.
9. (pregnan$ adj4 morbid$).af.
10. (pregnan$ adj4 mortalit$).af.
11. (maternal adj4 morbid$).af. or exp pregnancy outcome/
12. or/6-11
13. exp pregnancy complications/
14. exp delivery/
15. exp pregnancy/
16. exp puerperium/
17. exp Maternal Health Services/ or maternal welfare/
18. exp Hemorrhage/ and (pregnan$ or postpartum$ or post partum or post partal).mp.
19. exp ANALGESIA, OBSTETRICAL/
20. exp Uterine Rupture/
21. exp uterine hemorrhage/ or uterine inversion/ or exp uterine prolapse/ or exp uterine rupture/
22. exp Obstetric Surgical Procedures/
23. Endometritis/ and (pregnan$ or postpartum$ or post partum or post partal).mp.
24. exp pregnancy in diabetics/
25. exp HYPEREMESIS GRAVIDARUM/
26. or/13-25
27. exp Urinary Tract Infections/
28. exp MALARIA/
29. exp Bacteriuria/
30. exp RUBELLA/
31. exp Rectal Fistula/
32. exp anemia/
33. exp lung diseases/
34. exp vaginal fistula/
35. exp genital diseases, female/ or uterine prolapse/
36. exp Fecal Incontinence/
37. exp Urinary Incontinence/
38. exp Back Pain/
39. exp DEPRESSION/ or exp Depressive Disorder/
40. exp Stress Disorders, Traumatic/
41. exp "Wounds and Injuries"/
42. exp SUICIDE/
43. (pregnan$ or postpartum$ or post partum or post partal).mp.
44. (or/27-42) and 43
45. 44 or 26
46. 5 and 45
47. 12 or 46
48. limit 47 to human
49. Case Report/
50. 48 not 49
51. limit 50 to female
52. "1997".yr. and 51
53. "1998".yr. and 51
54. "1999".yr. and 51
55. "2000".yr. and 51
56. "2001".yr. and 51
57. "2002".yr. and 51

**EMBASE (Ovid):**

1. (maternal adj4 mortal$).af.

2. (maternal adj4 death$).af.

3. (maternal adj4 morbid$).af.

4. (pregnan$ adj4 mortal$).af.

5. (pregnan$ adj4 morbid$).af.

6. (pregnan$ adj4 death$).af.

7. or/1-6

8. exp Pregnancy Disorder/

9. exp Pregnancy/

10. exp Obstetric Care/

11. exp Childbirth/

12. exp Vagina Disease/

13. exp Uterus Prolapse/

14. exp Urinary Tract Infection/

15. *asymptomatic bacteriuria/ or *bacteriuria/

16. exp Malaria/

17. exp Urine Incontinence/

18. exp Toxoplasmosis/

19. exp Endometritis/

20. exp Rubella/

21. exp Rectum Fistula/

22. exp Feces Incontinence/

23. exp Anemia/

24. exp Depression/

25. exp Backache/

26. exp Suicide/

27. exp Lung Disease/

28. exp Injury/

29. exp Tetanus/

30. (pregnan$ or postpartum or post partum or postpartal).mp.

31. or/8-11

32. or/12-29

33. 30 and 32

34. exp Statistical Analysis/

35. exp "Population and Population Related Phenomena"/

36. exp comparative study/ or exp controlled study/

37. exp Register/

38. exp data analysis/ or *hospital information system/ or *medical information system/

39. exp Clinical Study/

40. exp Methodology/

41. *Health Survey/

42. exp Epidemiological Data/

43. exp Evaluation/

44. exp Maternal Welfare/

45. or/34-43

46. 31 or 33

47. 46 and 45

48. 7 or 44

49. 48 or 47

50. exp Case Report/

51. 49 not 50

52. limit 51 to (human and female)

**CINAHL (Ovid):**

1. mo.fs.
2. research/ or action research/ or exp administrative research/ or nursing administration research/ or analytic research/ or basic research/ or behavioral research/ or exp clinical research/ or comparative studies/ or descriptive research/ or ecological research/ or education research/ or exp epidemiological research/ or ethnographic research/ or ethnological research/ or ethnonursing research/ or exp evaluation research/ or exploratory research/ or field studies/ or "forecasting (research)"/ or exp health services research/ or exp historical research/ or methodological research/ or multimethod studies/ or needs assessment/ or physiological studies/ or pilot studies/ or policy studies/ or predictive research/ or replication studies/ or secondary analysis/ or survey research/ or trend studies/ or validation studies/ or exp research measurement/ or exp research methodology/ or exp research subjects/
3. exp POPULATION CHARACTERISTICS/
4. et.fs.
5. eh.fs.
6. ep.fs.
7. data analysis/
8. exp epidemiology/
9. exp statistics/
10. statistic$.mp.
11. statistic$.pt.
12. Tables$.pt.
13. or/1-12
14. (maternal adj4 mortal$).af.
15. (maternal adj4 death$).af.
16. (pregnan$ adj4 death$).af.
17. (pregnan$ adj4 morbid$).af.
18. (pregnan$ adj4 mortalit$).af.
19. (maternal adj4 morbid$).af.
20. exp Pregnancy Outcomes/
21. or/14-20
22. exp Pregnancy Complications/
23. exp "ANTEPARTUM/POSTPARTUM (OMAHA)"/
24. exp PREGNANCY/
25. exp PUERPERIUM/
26. exp Maternal Health Services/
27. exp Maternal Welfare/
28. exp hemorrhage/ and (pregnan$ or postpartum$ or post partum or post partal).mp.
29. exp ANALGESIA, OBSTETRICAL/
30. exp Uterine Hemorrhage/ and (pregnan$ or postpartum$ or post partum or post partal).mp.
31. exp Uterine Hemorrhage/
32. Postpartum Hemorrhage/
33. uterine inversion/
34. exp Uterine Prolapse/
35. Uterine Rupture/
36. exp Surgery, Obstetrical/
37. exp Endometritis/ and (pregnan$ or postpartum$ or post partum or post partal).mp.
38. Hyperemesis Gravidarum/
39. or/22-38
40. exp Urinary Tract Infections/
41. exp CYSTITIS/
42. exp MALARIA/
43. exp BACTERIURIA/
44. exp RUBELLA/
45. exp Rectal Fistula/
46. exp ANEMIA/
47. exp Lung Diseases/
48. exp Vaginal Fistula/
49. exp Genital Diseases, Female/
50. exp Fecal Incontinence/
51. exp Urinary Incontinence/
52. exp Back Pain/
53. exp DEPRESSION/
54. exp Stress Disorders, Post-Traumatic/
55. exp "Wounds and Injuries"/
56. exp Suicide/
57. (pregnan$ or postpartum$ or post partum or post partal).mp.
58. or/40-56
59. limit 58 to pregnancy
60. 58 and 57
61. 60 or 59
62. 61 or 39
63. (62 and 13) or limit 62 to evidence based practice
64. exp Animal Studies/
65. case study.pt.
66. 64 or 65
67. 63 not 66
68. 67 or 21
69. 68 and "1997".yr.
70. 68 and "1998".yr.
71. 68 and "1999".yr.
72. 68 and "2000".yr.
73. 68 and "2001".yr.
74. 68 and "2002".yr.
75. 68 and "2003".yr.

**CAB Abstract (Ovid):**

1. (maternal adj4 mortal$).af.
2. (maternal adj4 death$).af.
3. (pregnan$ adj4 death$).af.
4. (pregnan$ adj4 morbid$).af.
5. (pregnan$ adj4 mortalit$).af.
6. (maternal adj4 morbid$).af.
7. (pregnan$ adj4 outcome$).af.
8. (pregnan$ or postpartum$ or post partum$ or post partal$ or puerperium$).af.
9. miscarriage$.af.
10. (obstructed labo?r or abortion$).af.
11. maternal health$.af.
12. (pregnanc$ adj4 complication$).af.
13. prenatal$.af.
14. pre-natal$.af.
15. perinatal$.af.
16. post natal$.af.
17. postnatal$.af.
18. peri natal$.af.
19. or/1-18
20. exp birth/
21. exp pregnancy/
22. exp mothers/ or exp maternal behaviour/ or exp maternal effects/ or exp maternity/
23. exp abortion/ or exp fetal death/ or exp induced abortion/
24. exp contraception/ or exp family planning/ or exp population control/
25. exp Maternal Mortality/
26. exp PREGNANCY TOXAEMIA/ or exp PREGNANCY DIAGNOSIS/ or exp EXTRAUTERINE PREGNANCY/ or exp PREGNANCY COMPLICATIONS/ or exp EARLY PREGNANCY FACTOR/ or exp PREGNANCY/ or exp PREGNANCY RATE/
27. mortality.sh.
28. (20 or 21 or 22 or 24) and 27
29. 19 or 23 or 25 or 28
30. vv$.cc.
31. uu$.cc.
32. human.cw.
33. women.sh.
34. women.cw.
35. (29 and (30 or 31 or 32 or 33 or 34)) not exp Case Reports/
36. 35 not exp Case reports/
37. ll$.cc.
38. 36 not 37
39. "1997".yr. and 38
40. "1998".yr. and 38
41. "1999".yr. and 38
42. "2000".yr. and 38
43. "2001".yr. and 38
44. "2002".yr. and 38
45. "2003".yr. and 38

**Econlit (Ovid):**

1. (maternal adj4 mortal$).af.
2. (maternal adj4 death$).af.
3. (pregnan$ adj4 death$).af.
4. (pregnan$ adj4 morbid$).af.
5. (pregnan$ adj4 mortalit$).af.
6. (maternal adj4 morbid$).af.
7. (pregnan$ adj4 outcome$).af.
8. (pregnan$ or postpartum$ or post partum$ or post partal$ or puerperium$).af.
9. miscarriage$.af.
10. (obstructed labo?r or abortion$).af.
11. maternal health$.af.
12. (pregnanc$ adj4 complication$).af.
13. prenatal$.af.
14. pre-natal$.af.
15. perinatal$.af.
16. post natal$.af.
17. postnatal$.af.
18. peri natal$.af.
19. or/1-18
20. "1997".yr. and 19
21. "1998".yr. and 19
22. "1999".yr. and 19
23. "2000".yr. and 19
24. "2001".yr. and 19
25. "2002".yr. and 19
26. "2003".yr. and 19

**SocioFile (Ovid):**

1. (maternal adj4 mortal$).af.
2. (maternal adj4 death$).af.
3. (pregnan$ adj4 death$).af.
4. (pregnan$ adj4 morbid$).af.
5. (pregnan$ adj4 mortalit$).af.
6. (maternal adj4 morbid$).af.
7. (pregnan$ adj4 outcome$).af.
8. (pregnan$ or postpartum$ or post partum$ or post partal$ or puerperium$).af.
9. miscarriage$.af.
10. (obstructed labo?r or abortion$).af.
11. maternal health$.af.
12. (pregnanc$ adj4 complication$).af.
13. prenatal$.af.
14. pre-natal$.af.
15. perinatal$.af.
16. post natal$.af.
17. postnatal$.af.
18. peri natal$.af.
19. or/1-18
20. exp birth/ or exp abortion/ or exp birth control/ or exp birth order/ or exp birth spacing/ or exp fecundity/ or exp fertility/ or exp fertility decline/ or exp first birth timing/ or exp gynecology/ or exp illegitimacy/ or exp inbreeding/ or exp midwifery/ or exp pregnancy/ or exp prenatal care/ or exp prenatal testing/ or exp reproductive technologies/ or exp sexual reproduction/ or exp twins/
21. mothers/ or exp single mothers/ or exp working mothers/ or exp adolescent mothers/ or exp childrearing practices/ or exp wives/
22. exp mortality rates/ or death/ or exp demographic transition theory/ or fatalities/ or exp health/ or exp life tables/ or exp morbidity/
23. 20 or 21
24. 23 and 22
25. 24 or 19
26. "1997".yr. and 25
27. "1998".yr. and 25
28. "1999".yr. and 25
29. "2000".yr. and 25
30. "2001".yr. and 25
31. "2002".yr. and 25
32. "2003".yr. and 25

**LILACS:**

First run :

1. ex C13.703$ or Maternal mortalit$ or maternal morbidit$ or abortion$ or miscarriage$ or stillbirth$ or ectopic pregnan$ or obstructed labour [Subject descriptor] and
2. and 1998 [Country, year publication]

and not case report or animal [Limits]

Second Run :

1. ex C13.371.894.763$ or ex C15.378.071$ or HIV or SIDA or AIDS or infections or sexually transmitted diseases or sepsis or hepatitis or syphilis or tetanus or depression or malaria or fecal incontinence or urinary incontinence or haemorrhage or hemorrhage or eclampsia or preeclampsia or hypertensive disorders or pre eclampsia or violence or infertility or diabetes or anaesthesia complications [Subject descriptor]
2. and (Pregnancy or delivery or puerperium) [Limits]
3. and 1998 [Country, year publication]

**POPLINE**

Under keywords:

="Maternal Mortality" / ="Pregnancy Complications" / ="Maternal Health" / ="Abortion Induced" / (="Morbidity" & ="Mothers") / ((="Malaria" / ="AIDS" / ="Tetanus" / ="Sexually Transmitted Diseases" / ="Violence" / ="Depression" / ="Hemoglobin Level" / ="HIV Infections" / ="Reproductive Tract Infections" / ="Bleeding" / ="Anemia" / ="Hemoglobin Level Statistics" / ="Hemoglobin Level Women" / ="Fistula" / ="Hepatitis") & (="Pregnancy" / ="Puerperium" / ="pregnan* / ="delivery")) / ="Puerperal Disorders" / ="Pregnancy Outcomes"

**BIOSIS**

1. (reproductive system or reproductive system anatomy or reproductive system general methods or reproductive system pathology or "reproductive system physiology and biochemistry").cc. (384215)
2. (public health or public health environmental health miscellaneous or public health epidemiology or public health epidemiology communicable diseases or public health epidemiology miscellaneous or "public health epidemiology organic diseases and neoplasms" or "public health general and miscellaneous" or "public health health services and medical care" or "public health public health administration and statistics").cc. (243106)
3. 1 and 2 and female$.or. (5602)
4. (obstetrics or obstetrics c or obstetrics medical sciences human medicine psychiatry).mc. (14978)
5. 4 (14978)
6. (epidemiology or "epidemiology methods and techniques" or epidemiology primary).mc. (105098)
7. 4 or 5 (14978)
8. 7 and 6 (1499)
9. 3 or 8 (6196)
10. limit 9 to human (5744)
11. pregnan$.af. (71111)
12. childbirth$.af. (834)
13. matern$.af. (45370)
14. prenatal$.af. (15465)
15. (labour or labor).mp. (12728)
16. delivery.mp. (52502)
17. (postpartum$ or post partum$ or intra partum$ or intrapartum$).mp. (9663)
18. obstetric risk.af. (97)
19. child birth$.af. (103)
20. or/11-19 (158113)
21. 10 and 20 (2598)
22. limit 21 to yr=1997-2003 (2595)
23. maternal death$.af. (574)
24. (maternal mortal$ or maternal morbid$).af. (1125)
25. ((matern$ adj2 mortal$) or (matern$ adj2 morbid$)).mp. (1674)
26. (matern$ adj2 death$).mp. (735)
27. ((pregnan$ adj2 mortal$) or (pregnan$ adj2 morbid$)).mp. (691)
28. (pregnan$ adj2 death$).mp. (309)
29. or/23-28 (2835)
30. risk assessment.mp. (10230)
31. vital statistics.mp. (409)
32. morbidity.mp. (34917)
33. mortality.mp. (100294)
34. death.mp. (96492)
35. or/30-34 (204089)
36. 4 and 35 (2145)
37. (36 and 10) or 29 or 22 (5157)
38. limit 37 to human (4777)
39. 29 not 38 (380)
40. limit 39 to yr=1997-2003 (226)
41. [from 40 keep 10,29,32,37,61-62,64,76-77,100-102,126,145,148,150,153,162,170-173,180] (0)
42. 38 or 37 (5157)
43. (37 or 41) and (human$.tn. or human$.or.) (4784)
44. limit 43 to yr=1997 (349)
45. limit 43 to yr=1998 (726)
46. limit 43 to yr=1999 (803)
47. limit 43 to yr=2000 (630)
48. limit 43 to yr=2001 (547)
49. limit 43 to yr=2002 (589)
50. limit 43 to yr=2003 (1)
51. from 40 keep 3,10,13,25,30,39,53,58,65-66,68,80-81,91,106-109,133,137-138,150,154,157,159-160,163-164,183-185,193,219,226 (34)
52. limit 51 to yr=1997 (2)
53. limit 51 to yr=1998 (9)
54. limit 51 to yr=1999 (6)
55. limit 51 to yr=2000 (5)
56. limit 51 to yr=2001 (7)
57. limit 51 to yr=2002 (5)
58. 44 or 52 (351)
59. 45 or 53 (733)
60. 46 or 54 (809)
61. 47 or 55 (631)
62. 48 or 56 (554)
63. 49 or 57 (594)
64. from 58 keep 1-351 (351)
65. from 59 keep 1-733 (733)
66. from 60 keep 1-809 (809)
67. from 61 keep 1-631 (631)
68. from 62 keep 1-554 (554)
69. from 63 keep 1-594 (594)

**PAIS International**

1. (pregnancy complication$ or pregnancy ectopic$ or abortion$ or stillbirth$ or uterine rupture$ or abruptio placent$ or puerperal disorder$ or puerperal infection$ or eclampsia$ or pre-eclampsia$).af.
2. (maternal adj4 mortalit$).af.
3. (maternal adj4 death$).af.
4. (pregnan$ adj4 death$).af.
5. (pregnan$ or morbid$).af.
6. (materan$ or morbid$).af.
7. pregnancy outcome$.af.
8. (diabet$ or haemorrhage$ or hemorrhag$ or sepsis or malaria or fistula$ or anaemi$ or anemi$ or depression$ or hypertension$).af.
9. pregnan$.af.

8 and 9

1. 1 or 2 or 3 or 4 or 5 or 6 or 7 or 10
2. "1997".yr. and 11 (120)
3. "1998".yr. and 11 (136)

"1999".yr. and 11 (140)

1. "2000".yr. and 11 (140)
2. "2001".yr. and 11 (131)
3. "2002".yr. and 11 (107)
